# 11β-Hydroxyandrostenedione Returns to the Steroid Arena: Biosynthesis, Metabolism and Function

**DOI:** 10.3390/molecules181113228

**Published:** 2013-10-25

**Authors:** Liezl M. Bloem, Karl-Heinz Storbeck, Lindie Schloms, Amanda C. Swart

**Affiliations:** Department of Biochemistry, Stellenbosch University, Private Bag X1, Matieland 7602, South Africa; E-Mails: liezelb@sun.ac.za (L.M.B.); schloms@sun.ac.za (L.S.)

**Keywords:** adrenal H295R, androsterone, castration resistant prostate cancer (CRPC), cytochrome P450 11β-hydroxylase (CYP11B), hydroxysteroid dehydrogenase (HSD), 11keto-dihydrotestosterone (11KDHT), steroid 5α-reductase

## Abstract

The biological significance of 11β-hydroxyandrostenedione (11OHA4) has eluded researchers for the past six decades. It is now known that 11OHA4 is biosynthesized in the androgen arm of the adrenal steroidogenesis pathway and subsequently metabolized by steroidogenic enzymes *in vitro*, serving as precursor to recognized and novel androgenic steroids. These *in vitro* findings extend beyond the adrenal, suggesting that 11OHA4 could be metabolized in steroid-responsive peripheral tissues, as is the case for androgen precursor metabolites of adrenal origin. The significance thereof becomes apparent when considering that the metabolism of 11OHA4 in LNCaP androgen dependent prostate cancer cells yields androgenic steroid metabolites. It is thus possible that 11OHA4 may be metabolized to yield ligands for steroid receptors in not only the prostate but also in other steroid-responsive tissues. Future investigations of 11OHA4 may therefore characterize it as a vital steroid with far-reaching physiological consequences. An overview of the research on 11OHA4 since its identification in 1953 will be presented, with specific focus on the most recent works that have advanced our understanding of its biological role, thereby underscoring its relevance in health and disease.

## 1. Introduction

The biological significance of the C19 steroid 11β-hydroxyandrostenedione (11OHA4) has until very recently been overlooked, owing perhaps to the apparent lack of biological function and the uncertainty of its biosynthesis within the adrenal steroidogenic pathways. Early researchers were faced with the immense challenge of characterizing the intricacies of adrenal hormone production while having limited analytical tools. These analyses were further complicated by variations in steroid hormone secretion resulting from differences in age, gender and species as well as the overall complexity of the pathways which they were attempting to elucidate. Much of the research that has led to the characterization of the adrenal steroidogenic pathway, as it is understood today, was fuelled by investigations into excess adrenal androgen production and associated disease states, as well as the need to distinguish between adrenal and ovarian androgen production. The biosynthesis and metabolism of 11OHA4 was thus investigated in consequence of this research and a brief history of the metabolite will be presented within the context of these early investigations. This will be followed by an overview of the more recent works that led to the characterization of 11OHA4 as an important component of steroidogenesis.

## 2. The History of the 11OHA4 Metabolite

11OHA4 was first isolated from human adrenal incubates in 1955 by Touchstone *et al.* [1]. A previous study in 1953 by Jeanloz *et al.* [2] identified the metabolite in bovine adrenal glands following perfusion with androstenedione (A4) and identification using melting point analysis and infrared absorption spectra. Although the authors themselves did not detect 11-keto androstenedione (11KA4), they noted that the metabolite had been isolated from adrenal glands in previous studies. They therefore hypothesized that A4 was metabolized to 11KA4 via 11OHA4, disregarding previous suggestions that 11-ketosteroids were artefacts of isolation. A further steroid, 11β-hydroxy-5α-androstanedione (11OH-5α-dione), was also isolated following perfusion with A4.

**Scheme 1:**
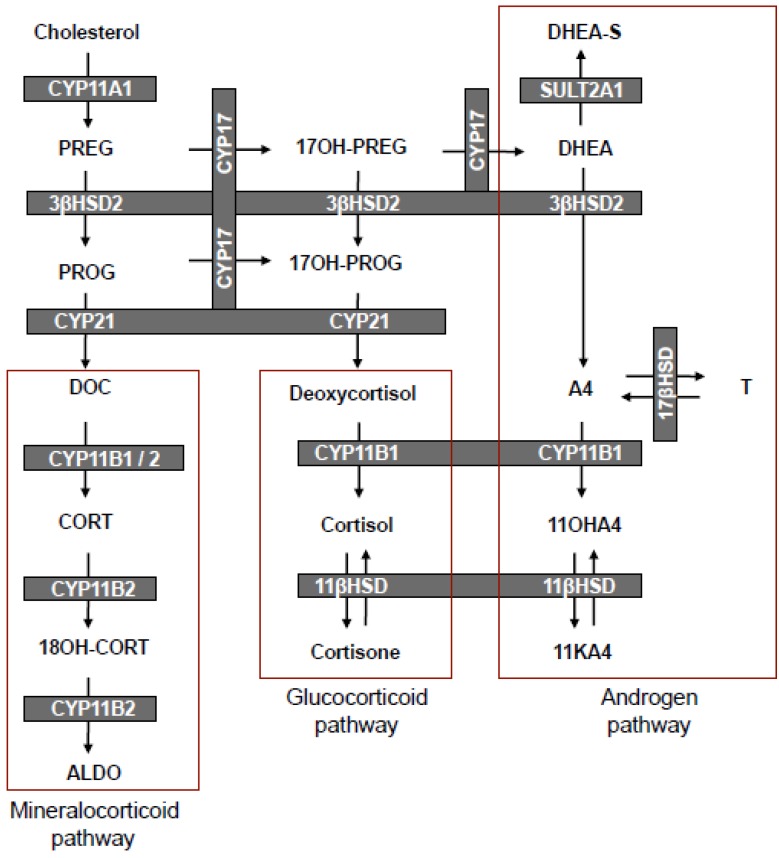
Steroid hormone biosynthesis in the mineralocorticoid, glucocorticoid and androgen pathways of the adrenal.

The first research that alluded to a divide between the glucocorticoid and androgen arms of the adrenal steroidogenic pathway, as it is known today (Scheme 1), came from the works of Dorfman [3] and Masuda [4] in the 1950s. Dorfman [3] observed from the analyses of urinary steroid metabolites that the C19 steroid (11KA4) and the C21 steroids (cortisol, cortisone and 21-desoxycortisone) gave rise to a common metabolite, 11β-hydroxyandrosterone (11OHAST), with the metabolism of the C19 steroid favouring the formation of the 5α-configuration of 11OHAST. He postulated that the C21 steroids may give rise to this metabolite by first undergoing a side-chain cleavage reaction, followed by the reduction of the double bond in ring A, or vice versa. In the first instance, cortisone and cortisol, for example, would form 11KA4 and 11OHA4, respectively, which in turn would give rise to predominantly the 5α-stereoisomers of 11-oxygenated 17-ketosteroids. This was, however, not the case with the C21 steroids yielding mainly 5β-stereoisomers, suggesting that the C21 steroids are first reduced and then subjected to a side-chain cleavage. Dorfman [3] therefore concluded that 11KA4, and perhaps 11OHA4, were the major precursors to the 5α-stereoisomers of the AST derivatives, 11KAST and 11OHAST; whilst AST formation was mainly ascribed to the metabolism of dehydroepiandrosterone (DHEA) and A4. He went on to suggest that the first metabolic step in the metabolism of DHEA would be the formation of A4 by the oxidation of the 3β-hydroxy group—a reaction now attributed to the activity of 3β-hydroxysteroid dehydrogenase type 2 (3βHSD2). Similarly, Masuda [4] observed that the C19 steroids, A4 and testosterone (T), as well as the C21 steroid, 17α-hydroxyprogesterone (17OH-PROG), gave rise to AST and its stereoisomer. The C19 steroids gave rise to both stereoisomeric configurations in approximately equal amounts; whilst 17OH-PROG favoured the formation of the 5β-stereoisomeric configuration. This led the author to conclude that A4, T and DHEA, rather than 17OH-PROG, accounted for AST secretion (5α-configuration). In addition, it was surmised that 11OHA4 and 11KA4 were the major precursors to 11OHAST [4].

In the following decade, Cohn and Mulrow [5] proposed a human adrenal steroidogenic pathway, very similar to the one currently accepted (Scheme 1), which demonstrated that A4 was primarily formed via the metabolism of pregnenolone (PREG) to 17α-hydroxypregnenolone (17OH-PREG) and subsequently to DHEA in the Δ^5^-pathway. Although their representation indicated deoxycortisol and cortisol as possible precursors of 11OHA4, their data did not support this notion, leaving the authors to suggest that 11OHA4 production could more likely be ascribed to the 11β-hydroxylation of A4. This was congruent with the later findings of Dadevoh *et al.* [6] who assayed the metabolism of radiolabeled progesterone (PROG) in human adrenals and detected negligible levels of 11OHA4. It was only later in 1993, that the contribution of 17OH-PROG in the Δ^4^-pathway to the biosynthesis of A4 in humans was shown to be negligible [7]. Goldzieher and Beerling [8] in 1969 evaluated urinary metabolites following the administration of radiolabeled 11OHA4, 11KA4 and cortisol. The authors observed that the C19 and C21 steroids generally favoured the formation of 5α- and 5β-stereoisomers of 11-oxygenated 17-ketosteroids, respectively, thereby confirming the earlier studies of Dorfman and Masuda [3,4]. They concluded that, although proof was lacking, 11OHA4 was generally considered to be the product of the 11β-hydroxylation of A4. Based on their own aforedescribed findings, together with those of other studies of adrenal vein blood analyses and *in vitro* adrenal tissue incubations, they also suggested that 11OHA4 appeared to be an important secretory product of the adrenal [8].

The Goldzieher group provided further support in favour of separate glucocorticoid and androgen biosynthesis pathways [9] and, by deduction, A4 as the likely precursor to 11OHA4 rather than cortisol. They suggested that cortisol could be reduced to its 5α-configuration allo-3α-tetrahydrocortisol (ATHF) – a metabolite not observed in the Dorfman [3] study, which in turn could be cleaved to form the same metabolites derived from 11OHA4, namely the 5α-stereoisomers of 11OHAST and 11KAST. Through the intravenous administration of radiolabeled ATHF to human test subjects, they observed that ATHF did not contribute significantly to the production of the relevant metabolites. After considering their findings within the context of other research, they concluded that cortisol, ATHF, 21-desoxycortisol and other cortisol-derived metabolites (tetrahydrocortisone (THE), tetrahydrocortisol (THF) and β-cortol), did not contribute significantly to the formation of 11OHAST, with the contribution of cortisol ranging from negligible to 7% of the detected radioactivity. Similarly, conversion assays of radiolabeled cortisol and A4 by means of *in vivo* perfusion of baboon adrenals as well as in minced adrenal tissue from humans and baboons, revealed that A4 was the predominant precursor of 11OHA4, with only low levels of 11OHA4 resulting from the side-chain cleavage of cortisol [10]. The first study to assay A4 metabolism in mitochondrial and microsomal fractions of bovine adrenals was performed in 1976, and showed that A4 was hydroxylated at a number of positions in the mitochondrial fraction, with the highest reaction rate for the hydroxylation at C11. Similar results were obtained for the microsomal fraction, although at lower reaction rates [11]. Noteworthy, metyrapone, used today as a specific inhibitor of cytochrome P450 11β-hydroxylase (CYP11B1), greatly inhibited the NADPH-dependent 11β-hydroxylation of A4. Attempts to characterize the enzymatic reactions in the adrenal steroidogenic pathway contributed, in part, towards the characterization of the cytochrome P450 enzymes as monooxygenases, a tale eloquently summarized by Estabrook [12].

The origin of 11OHA4 remained a topic of controversy despite convincing support, albeit deductive, favouring the 11β-hydroxylation of A4. Indeed, not all of the earlier research alluded to separate glucocorticoid and androgen pathways. Lombardo and Hudson [13] in 1959 observed the formation of 11OHA4, but not DHEA or A4, following incubation of human adrenal tissue slices with PREG as substrate. They therefore suggested that 11OHA4 did not arise from PREG but perhaps from the oxidative degradation of cortisol’s side-chain, or alternatively, the lyase of 17OH-PROG, followed by the hydroxylation of A4 in the Δ^4^-pathway. Hudson and Killinger [14] subsequently administered combinations of radiolabeled DHEA, PROG and cortisol to human adrenal homogenates. Although DHEA contributed significantly to 11OHA4 levels, the authors nevertheless concluded that cortisol could be a significant precursor to the adrenal production thereof. Adding to the confusion, Klein *et al.* [15] investigated the metabolism of radiolabeled steroids in human adrenal homogenates and demonstrated the conversion of deoxycortisol and deoxycorticosterone (DOC) to their respective hydroxylated products, as well as the conversion of deoxycortisol to 11OHA4 and A4, but not to cortisone. The first observations were seen in both the microsomal and mitochondrial fractions, whilst the cleavage of cortisol was only observed in the microsomal preparations, suggestive of 11β-hydroxylation and 17,20-lyase activity, respectively. The controversy was amplified by further reports of cortisol being converted to 11OHA4 in the microsomal, mitochondrial and supernatant fractions of adrenal preparations; and also by observations that 11OHA4 could be derived from T. Chang *et al.* [16] incubated human adrenal tissue homogenates with radiolabeled T and observed predominantly 11β-hydroxytestosterone (11OHT), together with low levels of A4, 11OHA4 and 11KA4, but no 5α-reduced metabolites. These findings were indicative of 17β-hydroxysteroid dehydrogenase (17βHSD) activity in human adrenals, with the authors suggesting that 11OHA4 likely arose from the dehydrogenation of 11OHT, or alternatively, the hydroxylation of A4. The topic of its origin, however, received little further attention, perhaps, in part, because 11OHA4 did not appear to be biologically significant. As consequence, its origin and potential adrenal androgenic pathways stemming from its metabolism were of little interest.

The low androgenicity of 11OHA4 had collectively been described by the works of Dorfman and Rosemburg *et al.* [17,18] in the 1960s. In both studies, steroids were applied directly to male White Leghorn chick’s combs once daily for 7 days. Comb and body weights were determined 24 h following the final application, allowing for the data to be expressed as comb ratios (comb (mg):body weight (g)). Rosemburg *et al.* [18] assessed the androgenicity of 11OHA4, along with its C9 bromo-, chloro- and fluoro- derivatives, as well as A4. Comparison of comb ratios revealed that A4 was the more androgenic metabolite. None of the steroids, however, matched the androgenicity of T, as determined by Dorfman [17], and it was subsequently postulated by Goldzieher *et al.* [19] in 1978 that the 11β-hydroxylation of A4 served as a biological mechanism to inactivate the metabolite, prohibiting its metabolism to T. Support for this hypothesis came 15 years later from Bélanger *et al.* [20] after observing the low androgenic potential of 11OHA4 in an androgen-sensitive *in vitro* model (35-fold less potent than DHT) and noting that Labrie *et al.* [21] had previously, in 1988, shown that A4 increased prostate weight and prostatic DHT levels in castrated rats. However, considered from a different angle, the 11β-hydroxylation of A4, and specifically the efficiency thereof, had been proposed to be an important regulator of adrenal androgen output [8]. These studies into the biological activity of 11OHA4 did not, however, ascribe function to the metabolite itself and the adrenal steroidogenic pathway, as it is generally accepted today, was first published in 1988 without 11OHA4, a testament to its apparent lack of physiological function [22].

As overviewed, the biosynthesis of 11OHA4 was shown to take place in the adrenal by either the lyase of cortisol or the hydroxylation of A4, with early studies favouring the latter. Although these earlier investigations reported the detection of 11OHA4 in human and bovine adrenal mitochondrial fractions, they did not directly ascribe the hydroxylation of A4 in humans to the activity of either the cytochrome P450 11β-hydroxylase enzymes, CYP11B1 and/or CYP11B2 (aldosterone synthase). It had, however, been shown that purified bovine P450 11β-hydroxylase catalyzed the hydroxylation of A4 at a rate approximately half that of DOC but higher than that of T [23]. A subsequent study using recombinant bovine CYP11B expressed in COS-1 cells, showed the conversion of A4 to 11OHA4 at a rate comparable to the conversion of DOC to corticosterone (CORT) [24]. It was later established that the human genome contained two CYP11B genes—one encoding CYP11B1, which exhibits 11β-hydroxylase activity only, and the other encoding CYP11B2, which also exhibits 11β-hydroxylase activity, but follows on to hydroxylate and oxidize C18 to form aldosterone (ALDO) [25,26]. Both CYP11B1 and CYP11B2 are primarily expressed in the adrenal cortex, where CYP11B1 catalyzes the 11β-hydroxylation of DOC and deoxycortisol to yield CORT and cortisol, respectively, whilst CYP11B2 catalyzes the conversion of DOC to ALDO. Interestingly, a single enzyme catalyzes these reactions in the bovine species [23]. As mentioned, the interest in 11OHA4 had dwindled and the metabolite was not included in subsequent studies conducted regarding the catalytic activity and substrate preferences of these two enzymes.

Subsequent to these investigations, 11OHA4 had been shown to be one of the major metabolites in H295R cells, an adrenal cell model, under conditions mimicking stimulation by adrenocorticotrophic hormone (ACTH). The H295R cells are capable of producing all the steroids of the three adrenal cortex zones including the mineralocorticoids, glucocorticoids and adrenal androgen precursors. These cells are, however, insensitive to ACTH and forskolin is generally used as an inducer of steroidogenesis [27]. Recent investigations reported that ACTH stimulated the production of 11OHA4 in primary adrenal cultures and that the metabolite was indeed one of the major steroids produced by the human adrenal [27], a finding corroborated in H295R cells by the quantification of 11OHA4 [28]—collectively renewing interest in this forgotten metabolite.

## 3. The Biosynthesis of 11OHA4

Significant advances in analytical techniques have enabled the accurate detection and quantification of various adrenal steroid metabolites, which previously proved to be challenging. The analyses of specific end products or intermediates had only been possible using radioimmunoassay (RIA) and enzyme-linked immunosorbent assay (ELISA) techniques, with conventional HPLC and derivitization also only allowing detection of a limited number of steroids. Exemplifying these advances are the aforementioned studies by Xing *et al.* [27] and Schloms *et al.* [28] having employed liquid chromatography/tandem mass spectrometry (LC-MS/MS) and ultra-performance liquid chromatography-MS/MS (UPLC-MS/MS), respectively. The latter study allowed for the detection and quantification of steroid intermediates and end products in a single chromatographic step without the need for derivitization. These studies confirmed that 11OHA4 is a major product of adrenal steroidogenesis. UPLC-MS/MS analyses of C19 steroids and their precursor metabolites in H295R cells (Table 1) showed significantly increased levels of the steroids in the Δ^5^-pathway, PREG and DHEA, upon forskolin stimulation. In both basal and stimulated cells, A4 was the major C19 steroid produced, with the subsequent production of 11OHA4 being higher than T and 11KA4. A significant increase in 11OHA4, in contrast to T and 11KA4, was also observed upon stimulation which was not unexpected, since forskolin upregulates CYP11B1 mRNA and cytochrome P450 17α-hydroxylase/17–20 lyase (CYP17A1) in H295R cells [29,30,31], thus increasing precursor steroid levels, particularly evident in A4 production. Forskolin stimulation had no significant effect on the levels of T, while 11OHT and 11KT remained undetectable [28]. These findings were in agreement with a recent report, which showed that ACTH did not increase T levels or stimulate either 17βHSD type 3 or type 5 (AKR1C3) activity in normal adrenal cells [32].

**Table 1:** UPLC-MS/MS analyses of C19 steroids produced in H295R cells. Adapted from [33].

Steroid metabolite	Basal	+ Forskolin			
	Total ± SEM (nM)	Total ± SEM (nM)	Fold Change		
PREG	237.7 ± 12.7	799.5 ± 34.2	↑	3.4	***
17OH-PREG	ND	ND			
DHEA	232.3 ± 18.2	464.5 ± 34.7	↑	2.0	***
DHEA-S	3.4 ± 0.2	5.1 ± 0.3	↑	1.6	***
A4	913.2 ± 29.2	1338.0 ± 81.1	↑	1.4	***
11OHA4	100.4 ± 5.2	329.9 ± 27.2	↑	3.5	***
11KA4	2.8 ± 1.0	3.4 ± 0.8			
T	46.0 ± 3.8	50.0 ± 2.4			
11OHT	ND	ND			
11KT	ND	ND			

**Figure 1:**
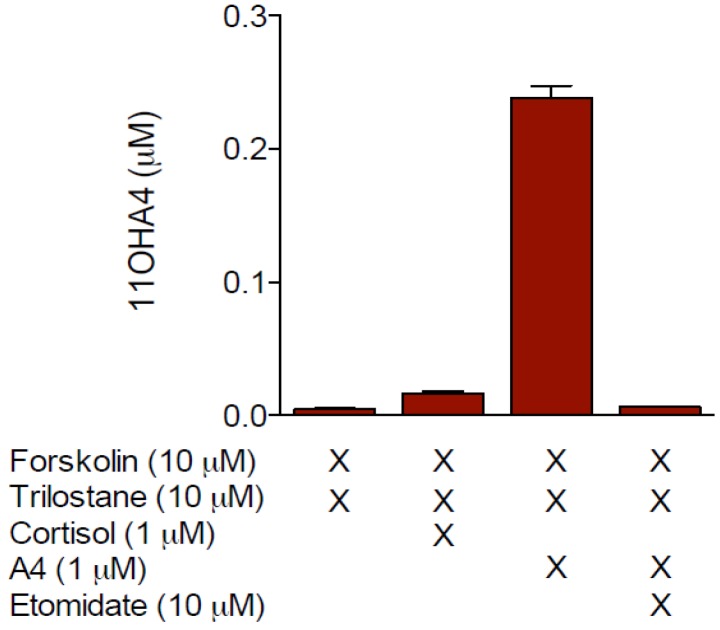
Analysis of 11OHA4 production in H295R cells.

The potential activity of human CYP17A1 towards both cortisol and deoxycortisol, which would upon cleavage of the side chain, produce either 11OHA4 or A4 respectively, did not yield either of the products (unpublished data). This finding was confirmed in H295R cells treated with trilostane, a selective inhibitor of 3βHSD, which prohibits the metabolism of PREG, 17OH-PREG and DHEA by the enzyme. No 11OHA4 was detected in these cells after the addition of cortisol both with and without forskolin treatment (Figure 1).

Conversely, 11OHA4 was detected upon administering A4 to forskolin-stimulated cells, in the presence of trilostane. The inclusion of the CYP11B1 inhibitor, etomidate, abolished 11OHA4 formation, clearly indicating that 11OHA4 was the product of the 11β-hydroxylation of A4 by CYP11B1 [28]. This correlated well with earlier data from Liakos *et al.* [30], who showed that transforming growth factor β1 (TGFβ1) inhibited the expression of CYP11B1 and CYP11B2 in H295R cells, resulting in decreased levels of ALDO, cortisol as well as 11OHA4. Interestingly, the administration of labeled CORT, cortisol and A4 in guinea-pig glomerulosa-fasciculata cells showed A4 to be the sole precursor of 11OHA4, thereby providing compelling support for the dismissal of cortisol as a potential precursor, at least in the adrenal [34]. Indeed, more recent works have proposed that the lyase of cortisol to form 11OHA4 occurs in peripheral tissues [35,36].

**Figure 2:**
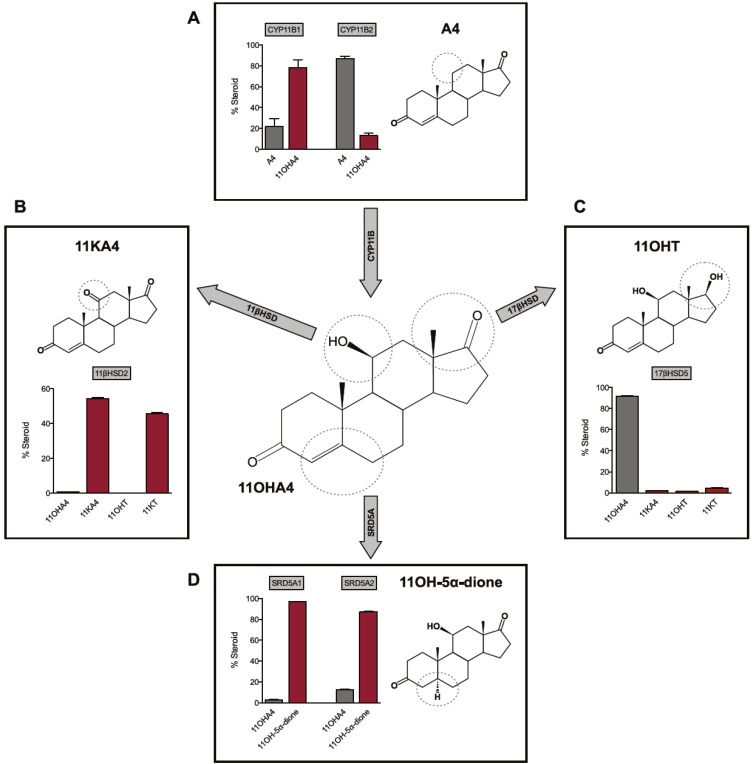
Schematic representation of the biosynthesis and metabolism of 11OHA4. Adapted from [33,37].

In our aforedescribed studies we also observed the conversion of T to 11OHT in forskolin-stimulated cells, in the presence of trilostane [28]. Upon addition of etomidate, however, low levels of 11OHT were nevertheless detected, indicating the possible involvement of CYP11B2. We recently reported that human CYP11B1 and CYP11B2 are both capable of catalyzing the 11β-hydroxylation of A4 and T to yield 11OHA4 and 11OHT, respectively, in transiently transfected CHO-K1 and COS-1 cells. Our data showed that CYP11B1 readily catalyzed the conversion of A4 to 11OHA4, in contrast to the minimal conversion by CYP11B2 following an extended incubation period (Figure 2A) [28,33]. The 11β-hydroxylation of T was catalyzed to the same degree by both CYP11B1 and CYP11B2. Upon conversion of A4 in COS-1 cells expressing CYP11B1 and CYP11B2, we also detected low levels of 11KA4 as a result of endogenous 11β-hydroxysteroid dehydrogenase type 2 (11βHSD2) expression [25], prompting further investigations into the activity of 11β-hydroxysteroid dehydrogenases.

## 4. The Metabolism of 11OHA4

### 4.1. 11β-Hydroxysteroid Dehydrogenase (11βHSD)

The 11β-hydroxysteroid dehydrogenase type 1 (11βHSD1) and 11βHSD2 enzymes catalyze the inter-conversion of active 11-hydroxy glucocorticoids (cortisol and CORT) to their inactive 11-keto forms (cortisone and 11-dehydrocorticosterone). In addressing our previous observation of the dehydrogenation of 11OHA4 to 11KA4 via the activity of 11βHSD2, we reported that the introduction of a hydroxyl group at the C11 position of A4 or T, by either CYP11B1 or CYP11B2, yields substrates for both the 11βHSD isozymes [33]. More specifically, we observed that 11βHSD1 catalyzed the conversion of 11KA4 to 11OHA4, whilst 11βHSD2 catalyzed the reverse reaction (Figure 2B). In addition, these enzymes were also able to inter-convert 11KT and 11OHT. Interestingly, we also detected additional products in our CHO-K1 conversion assays, other than those of the 11βHSD catalyzed reactions, suggestive of endogenous 17βHSD activity in this cell line. These findings indicated that 17βHSD may also metabolize certain hydroxy- and keto-derivitives of A4 and T as had already been suggested by Chang *et al.* [16] in 1963.

### 4.2. 17β-Hydroxysteroid Dehydrogenase (17βHSD)

The 17βHSD enzymes inter-convert the 17-hydroxyl and 17-keto groups of androgens (C19) and estrogens (C18). Our investigations into the activity of 17βHSD3 and 17βHSD5, the most prominent enzymes in the conversion from A4 to T [38], showed that while both readily converted 11KA4 to 11KT, these enzymes exhibited negligible activity towards 11OHA4 (Figure 2C). Our investigations to date into the reductive/oxidative activites of different 17βHSDs collectively indicated that the reductive activity thereof favours a keto-group at C11. The endogenous 17βHSD2 activity in COS-1 cells, which functions as a dehydrogenase, however, favoured a hydroxy-group at C11 [37].

### 4.3. Steroid 5α-Reductase (SRD5A)

**Figure 3:**
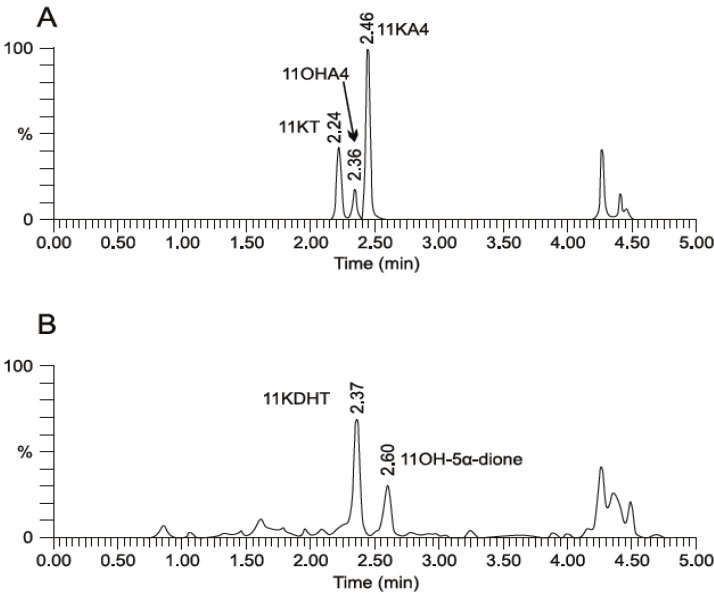
UPLC–MS/MS analysis of (**A**) 11OHA4 and (**B**) 11OH-5α-dione metabolism by 11βHSD2 and 17βHSD in LNCaP cells. Adapted from [37].

The steroid 5α-reductase isozymes, SRD5A1 and SRD5A2, catalyze the 5α-reduction of an array of C19 and C21 steroids which contain the Δ^4^ 3-keto moiety. One of the most important and well documented roles of SRD5A is the localised conversion of circulating T within target tissue to its 5α-reduced form DHT, which serves as the most potent natural androgen [39,40]. SRD5A also plays a vital role, together with 17βHSD, in the metabolism of the seemingly weak adrenal androgen A4 to DHT in target tissue via the 5α-dione pathway [41]. We therefore hypothesized that 11OHA4, which has negligible androgenicity, and its potentially androgenic derivatives could similarly contribute to the androgen pool in undergoing reduction by SRD5A. In addressing this hypothesis, we showed that both SRD5A1 and SRD5A2 catalyzed the 5α-reduction of 11OHA4 to form 11OH-5α-dione (Figure 3d). In the same investigation, we also reported that 11KA4, 11OHT and 11KT were converted to 11-keto-5α-androstanedione (11K-5α-dione), 5α-dihydro-11β-hydroxytestosterone (11OHDHT) and 5α-dihydro-11-keto-testosterone (11KDHT)—novel C19 steroids which had not previously been described [37]. Interestingly, 11OH-5α-dione was detected in the early works of Jeanloz *et al.* [2] following perfusion of bovine adrenals with A4, however, this metabolite has since received scant attention.

## 5. The Metabolism of 11OHA4 *in Vivo*

Our data clearly showed that 11OHA4 and its derivatives, 11KA4, 11OHT and 11KT, are metabolized by 11βHSD, 17βHSD and SRD5A to 11OH-5α-dione and the novel steroids, 11K-5α-dione, 11OHDHT and 11KDHT. We subsequently reported that the metabolism of 11OHA4 led to the production of predominantly 11KDHT via a number of C19 intermediates, including 11KA4 and 11KT (Figure 3) in the androgen dependent prostate cell line, LNCaP, in which the relevant enzymes are expressed [37]. In addition to identifying novel steroids, our conversion assays in LNCaP cells confirmed that these steroids indeed serve as substrates for 11βHSD, 17βHSD and SRD5A. This finding implicates the existence of novel steroidogenic pathways in peripheral tissues expressing the relevant enzymes and proposes a potential role for this unique adrenal steroid hormone.

The metabolism of 11OHA4 to these novel androgens in peripheral tissue is indeed likely considering the adrenal output of 11OHA4 and the tissue specific expression of the relevant enzymes. As previously mentioned, 11OHA4 is a major product of the adrenal and its output is elevated in response to ACTH [27,28]. CYP11B1 and CYP11B2, considered to be primarily adrenal enzymes, were shown to readily hydroxylate both A4 and T, however, with low adrenal output of 11OHT possibly due to low T levels in the adrenal which could be attributed to low levels of 17βHSD expression, as supported by the microarray analyses of steroidogenic enzymes in human adrenal glands conducted by Rege *et al.* [42]. The 11-keto-derivatives of A4 and T also showed low adrenal output reflective of low levels of 11βHSD2. The expression of the latter has, however, been demonstrated in the androgen dependent prostate cancer cell line, LNCaP [43,44]. Conversely, the expression of 11βHSD1 in the prostate remains questionable, with its primary expression in the liver, bone and adipose tissue [43,45]. The activity of 17βHSD observed in our *in vitro* findings is probable in peripheral tissue with 12 different 17βHSD enzymes having been characterized to date. While 17βHSD enzymes differ regarding cofactors and substrates, they are widely expressed and catalyze the inter-conversion of the hydroxyl and keto moeities at C17 of androgens and estrogens, thus modulating the biological potency of steroids towards their respective receptors, with the keto-forms being less potent than the hydroxy-forms [38]. It is, however, the 5α-reductases which lead to the production of active androgens and notably, the three known 5α-reductase isozymes are widely expressed [46,47]. Within the prostate, SRD5A1 and SRD5A2 are responsible for converting gonadal T to its 5α-reduced form, DHT [39,40]. SRD5A also plays a vital role in the “alternate 5α-dione pathway”, which results in the intratumoral production of DHT from the adrenal steroids, A4 and DHEA(S), while bypassing T entirely [41,48]. Instead, A4 is reduced by SRD5A to 5α-dione, followed by the 17-keto reduction to DHT. Furthermore, A4 is preferred as a substrate of SRD5A1 over T [41,49]. It is therefore clear that the adrenal contributes precursors to the androgen pool in hormone dependent tissues and tumours.

## 6. The Biological Significance of 11OHA4

As in the case of A4 and DHEA, the peripheral metabolism of 11OHA4 yields androgenic products from a non-androgenic substrate. Our research showed that 11OHA4 did not exhibit detectable androgenic activity and that the androgenicity of 11KA4 was similar to that of A4. The 5α-reduction of 11OHT and 11KT to 11OHDHT and 11KDHT, respectively, resulted in significant increases in androgenic activity at a physiologically relevant concentration, with 11KDHT acting as a full agonist comparable to DHT (Figure 5). The androgenicity of 11OHT was 2-fold less than that of T and 11KT [37]. These findings were consistent with those from a previous study, showing that 11KT elicits an androgenic response comparable to that of T, whilst 11OHT shows relatively weaker activity [50]. These data suggest that 11KDHT, in particular, represents a novel androgen which may play an important role in driving AR-mediated gene expression.

**Scheme 2:**
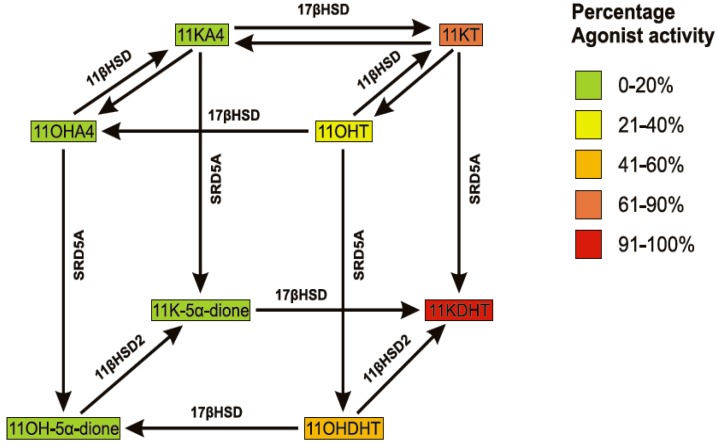
Schematic representation of the conversion of 11OHA4 to 11KDHT catalyzed by 11βHSD, 17βHSD and SRD5A. AR agonist activity of steroids is relative to DHT.

There are three potential metabolic routes from 11OHA4 to 11KDHT, indicated in Scheme 2, with the preferred route depending on the kinetic characteristics of each enzyme together with their relative expression levels in target tissues. The metabolism of 11OHA4 therefore offers a robust mechanism for the production of known and novel C19 steroids, which not only have a potential role in normal steroidogenic tissue expressing enzymes highlighted in this review, but also within the context of disease. One such example can be found in castration resistant prostate cancer (CRPC). The primary treatment of androgen dependent prostate cancers is the inhibition of testicular T, which is known as androgen deprivation therapy. While initially effective, many cases progress to CRPC over time [51,52]. At this point it should be reemphasized that while T is the major androgen found in circulation of healthy males, it is in fact its reduced form, DHT, which is the most potent androgen. The intratumoral levels of DHT in CRPC, following androgen deprivation, is sufficient to activate the AR [53] and is derived from the aforementioned “alternate 5α-dione pathway” in which the adrenal steroids A4 and DHEA(S) are converted to DHT via 5α-dione [41,48]. Our research to date suggests that the metabolism of 11OHA4 (Figure 3), as demonstrated in LNCaP cells, yields novel androgens such as 11KDHT, which may contribute to the activation of the AR in CRPC.

While the “alternate 5α-dione pathway” leads to DHT production, the intermediate, 5α-dione, also serves as substrate for the 3α-hydroxysteroid dehydrogenases (3α-HSD) resulting in the formation of AST. It is thus possible that 11OHA4, once reduced, may follow the same metabolic route. Interestingly, 11OHAST and 11KAST, the proposed downstream metabolites of 11OH-5α-dione and 11K-5α-dione, respectively, had already been identified in 1954 in the works of Dorfman [3] who suggested that 11OHA4 and 11KA4 were the precursors of these metabolites. Our findings may also extend to other peripheral tissue expressing the necessary enzyme systems. The skin is one such an example, as was recently shown by Slominski *et al.* [54] who noted that skin cells contain “the entire biochemical apparatus necessary for the production of glucocorticoids, androgens and estrogens”. The cutaneous steroidogenic pathway is capable of producing a number of active steroids including the androgens T and DHT. The authors further discuss the role of sex hormones in skin disorders, implicating increased AR levels, 17βHSD3 and 17βHSD5 in conditions ranging from balding scalp to acne.

## 7. Summary and Conclusions

In the mammalian adrenal, the expression of CYP11B1, together with low levels of 17βHSD5 and 11βHSD2, results in the production of 11OHA4, 11KA4, 11OHT and 11KT [33,42,55]. Of these steroids, 11OHA4 showed no detectable androgenic activity, while 11KA4 demonstrated androgenic activity similar to that of A4. 11OHT and 11KT were even more potent, having similar androgenic activity to that of T. The levels of 11KA4, 11OHT and 11KT produced by the adrenal are, however, more than 150-fold lower than that of 11OHA4 [42]. Given the fact that 11KA4, 11OHT and 11KT are all more androgenic than 11OHA4, it is therefore not inconceivable that given the necessary enzymatic machinery, 11OHA4 can be the substrate for the production of active androgens in the peripheral target tissue of mammals. One only needs to look at the role of the adrenal androgen precursors, A4 and DHEA(S), in CRPC for evidence of this. While A4 and DHEA(S) are both considered to be weak androgens, they have both been implicated in driving CRPC, which is androgen dependent, in the absence of T, the primary circulating androgen.

Our findings thus far confirm that while 11OHA4, a steroid unique to the adrenal, exhibits negligible androgenic activity, its metabolism gives rise to distinct steroid pathways leading to the formation of novel androgens. The 11OHA4 derivatives could therefore serve as an additional source of adrenal derived androgens, other than DHEA(S) and A4, which may significantly contribute to CRPC through the activation of the AR. Furthermore, 11OHA4 derivatives and perhaps even 11OHA4 itself, may interact with other steroid receptors in peripheral tissue, endocrine organs and skin. The exact consequences of 11OHA4 and its derivatives remain to be defined. What has, however, been established with certainty is that the metabolite 11OHA4, disregarded for decades, has reemerged as a vital component of the adrenal steroidogenic pathway and as a precursor to known and novel androgenic steroids which may have significant implications in health and disease.

## Supplementary Materials

Supplementary materials can be accessed at: http://www.mdpi.com/1420-3049/18/11/13228/s1.
