# Supplementary material for: 11β-Hydroxyandrostenedione Returns to the Steroid Arena: Biosynthesis, Metabolism and Function
Source: Molecules. 2013 Oct 25;18(11):13228–44. doi: 10.3390/molecules181113228 (PMC6270415; doi:10.3390/molecules181113228)
Supplement: Supplementary file 1 [file molecules-18-13228-s001.pdf]

Supplementary Materials

Table S1. Summary of the investigations into the origin of 11OHA4.

Tissue/specimen	Administered	Steroid isolated	Hypotheses/Conclusions	Reference
Bovine adrenals (perfusion)	A4	11OHA4;<br>11OH-5α-dione	• A4→11OHA4→11KA4	[2]
Human urinary samples	11KA4 (C19)<br>Cortisol (C21)<br>Cortisone (C21)<br>21-desoxycortisone (C21)	11OHAST:5α > 5β<br>11OHAST:5α < 5β<br>11OHAST:5α < 5β<br>11OHAST:5α < 5β	• C19 steroids→ primarily 5α-stereoisomere of 11OHAST<br>• C21 steroids→ primarily 5β-stereoisomere of 11OHAST	[3]
Human adrenals	Corticotropin	11OHA4	• 11OHA4 is produced in the human adrenal	[1]
Human urinary samples	A4 (C19)<br>T (C19)<br>DHEA (C19)<br>17OH-PROG (C21)	AST:5α = 5β<br>AST:5α = 5β<br>AST:5α = 5β<br>AST:5α < 5β	• C19 steroids (and not C21)→5α-stereoisomere of AST<br>• 11KA4, 11OHA4→primarily 11OHAST	[4]
Human adrenals	PREG	11OHA4 (no A4 or DHEA)	• Cortisol→11OHA4<br>• 17OH-PROG→ A4→11OHA4	[13]
Human adrenals	Radiolabeled PROG, DHEA, 17OH-PREG	A4; 11OHA4, DHEA	• PREG→ 17OH-PREG→DHEA→A4 (Δ^5-pathway)<br>• A4→11OHA4<br>• Deoxycortisol, cortisol↛ 11OHA4	[5]
Human adrenals	Radiolabeled T	A4; 11KA4; 11OHA4; 11OHT	• A4→11OHA4<br>• 11OHT→11OHA4	[16]
Human adrenals	Radiolabeled PROG	11OHA4	• PROG→ negligible 11OHA4	[6]

Table S1. *Cont.*

Tissue/specimen	Administered	Steroid isolated		Hypotheses/Conclusions	Reference
Urinary samples	Radiolabeled 11OHA4	11-oxy-17-ketosteroids	5α > 5β	• Primarily C19 steroids→5α-stereoisomeres of 11-oxygenated 17-ketosteroids	[8]
	Radiolabeled 11KA4		5α > 5β		
	Radiolabeled cortisol		5α < 5β		
Urinary samples	Radiolabeled ATHF (5α-derivative of cortisol)	11OHAST; 11KAST		• ATHF→negligible 5α-stereoisomeres	[9]
Human adrenals	Radiolabeled DHEA, PROG, cortisol (in combinations)	11OHA4		• Primarily DHEA→11OHA4<br>• Cortisol→11OHA4	[14]
Baboon adrenals (perfusion); human and baboon adrenals	A4	11OHA4		• Primarily A4→11OHA4	[10]
	Cortisol	Low levels of 11OHA4			
Human adrenals: microsomal and mitochondrial fractions	Radiolabeled deoxycortisol	Cortisol; A4; 11OHA4 (no cortisone)		• Cortisol cleavage in only the microsomal fraction	[15]
	Radiolabeled DOC	CORT			
COS-1 cells (transfected with human CYP17A1)	PROG	17OH-PROG (no A4)		• PROG, 17OH-PROG→A4	[7]
	PREG	17OH-PREG; DHEA			

5α-5alpha, 5β-5beta, A4-androstenedione, AST-androsterone, ATHF-allo-3α-tetrahydrocortisol, CORT-corticosterone, CYP17A1-cytochrome P450 17α-hydroxylase/17-20 lyase, DHEA-dehydroepiandrosterone, DOC-deoxycorticosterone, PREG-pregnenolone, PROG-progesterone, 11KAST-11β-ketoandrosterone, 11KA4-11-ketoandrostenedione, 11OHAST-11β-hydroxyandrosterone, 11OHA4-11β-hydroxyandrostenedione, 11OH-5α-dione-11β-hydroxy-5α-androstanedione, 11OHT-11β-hydroxytestosterone, 17OH-PREG-17α-hydroxypregnenolone, 17OH-PROG-17α-hydroxyprogesterone.
